# Pancreatic cancer, stroma, and exosomes

**DOI:** 10.1007/s13105-022-00898-1

**Published:** 2022-05-30

**Authors:** Daniel Closa

**Affiliations:** Dept. Experimental Pathology, IIBB-CSIC-IDIBAPS, Barcelona, Spain

**Keywords:** Pancreatic cancer, Stroma, Exosomes, CAF, TAM, Extracellular vesicles

## Abstract

In the pathogenesis of pancreatic adenocarcinoma, tumor stroma plays a key role in both aggressiveness, immune evasion, resistance to chemotherapy, and the ability to metastasize. Among the elements that characterize the behavior of the stroma, extracellular vesicles and, in particular, exosomes play an important role. These extracellular vesicles carry a wide range of bioactive molecules, from transcription factors to microRNAs, which can substantially alter the phenotype of the cellular components of the stroma. Exosomes are involved in the exchange of signals between tumor cells, tumor-associated macrophages, cancer-associated fibroblasts, and also with the healthy cells surrounding the tumor. They can transfer resistance to chemotherapeutic drugs, promote the epithelial-mesenchymal transition, modify the phenotype of macrophages, or induce the expression of molecules that alter the extracellular matrix to facilitate migration and metastasis. On the other hand, all these characteristics make these vesicles first-rate therapeutic targets, as controlling their functionality could greatly enhance the effectiveness of treatments that, today, are still far from be satisfactory.

## Introduction

Pancreatic ductal adenocarcinoma (PDAC) is one of the deadliest solid tumors, with a 5-year survival rate of lower than 8% [32]. This poor prognosis is due, in large part, to a number of factors that, separately, would already be a problem but that combined make this type of cancer a formidable therapeutic challenge. On the one hand, there is the lack of specific biomarkers, also the fact that the associated symptoms are very unspecific, and finally, it is a type of cancer that has a huge capacity to metastasize even in the very early stages of the process. All this explains why the percentage of patients who are candidates for surgical resection is particularly low. On the other hand, it is becoming increasingly clear that one of the factors contributing to its aggressiveness is the dense stroma surrounding the tumor. In fact, the presence of a large stromal component is one of the characteristics of PDAC. [33]. This desmoplastic stroma consists of stromal cells, mainly cancer-associated fibroblasts (CAF) but also endothelial and immune cells, and extracellular matrix, a dense and heterogeneous network made of collagen, elastin, fibronectin, and different sulfated glycosaminoglycans. This microenvironment plays a relevant role in the immune evasion from the host antitumor immune system and also induces chemotherapeutic resistance through different mechanisms [25].

The maintenance of the structure of the stroma and the changes it undergoes as it adapts to the progression of the disease or to the different treatments are based on a complex molecular network of signal exchange between the stromal cells and tumor cells, but also on the surrounding healthy cells. A number of lines of research are focused on the modulation of mediators such as connective tissue growth factor [38], focal adhesion kinase [44], secreted protein acidic and rich in cysteine [26], or hyaluronic acid [43]. However, in recent years, the role of extracellular vesicles, and in particular exosomes, as communication systems involved in the different processes linked to tumor stroma, has gained much importance.

Exosomes are extracellular vesicles that can carry a wide range of proteins, nucleic acids, and other mediators [40], making them an excellent system for transmitting complex messages between cells [13], between distant tissues [6], or, also, between tumor and stroma [14]. Its role in cancer is being studied extensively, and we know now that they play a role in almost all the processes involved in the tumor progression and forming premetastatic niches during cancer development to distant sites. Exosomes released by tumor cells could transfer oncogenic molecules [1], promote anti-apoptotic effects [39], trigger angiogenesis [55], or modify cell metabolism [57], thus contributing to cancer growth. They also promote the formation of metastasis by different mechanisms. Brain cancer cells could invade distant organs due to the ability of exosomes to modify the blood–brain barrier [60], the particular integrin profile of exosomes appears to regulate metastatic organotropism in different cancers [18], and in melanoma, exosomes promote the conditioning of lymph nodes facilitating the lymphatic metastasis [17]. This enormous versatility makes it difficult to understand the functions of the stroma in PDAC without considering the role played by exosomes generated by tumor cells, stroma cells, and also healthy cells surrounding the tumor.

## Exosomes

Exosomes are small extracellular vesicles (50–160 nm in diameter) and a lipid bilayer membrane originated through the endosomal pathway by, virtually, all cells [23]. This last fact is related to one of the problems that researchers face when determining the effects of these vesicles: their extremely high heterogeneity [56]. This means that in any sample obtained from biological fluids, exosomes from multiple cellular sources are mixed, so the analysis of their charge must be carefully interpreted [47]. Obviously, the content of the exosomes will determine the effects they induce on the recipient cells.

There are a number of proteins related to the synthesis of exosomes and the transport and fusion of membranes that are considered characteristics of exosomes. This includes annexins, Rab GTPases, ALIX, or TSG101. Exosomes also tend to carry heat shock proteins, adhesion molecules, nuclear enzymes, signal transducers, and proteins involved in metabolism [56]. However, it has been described that exosomes associated with cancer and, in particular, pancreatic cancer have enrichment in proteins related to tumorigenesis, metastasis, and cancer-specific signaling pathways [15].

The other relevant components of exosome cargo are the nucleic acids. Although the first nucleic acids identified in exosomes were miR and mRNA, it was soon seen that they can also carry tRNAs, lncRNA, and even fragments of genomic double-stranded DNA. The role of microRNAs has been of particular interest as it has been linked to features such as transfer of resistance to treatments [8, 51], acquisition of an anti-inflammatory phenotype in macrophages [45], or epithelial-mesenchymal transition [29]. On the other hand, mRNA carried by exosomes also could contribute to the characteristics of cancer fibroblasts due to the transfer of mRNA for COL5A1, α-SMA, or hTERT [11, 30]. Finally, large fragments of double-stranded DNA with mutated KRAS and TP53 have been detected in the plasma exosomes of pancreatic cancer patients [22]. A fact with relevant consequences for the detection and diagnosis of PDAC.

Once released into the intercellular medium, exosomes can be captured by target cells through different mechanisms that depend on the type and status of the recipient cell. Capture and uptake may occur by phagocytosis, macropinocytosis, through a specific, receptor-dependent pathway or by both clathrin-dependent and clathrin-independent pathways [31]. Contradictory results have been reported in these processes, and it is clear that the mechanisms of targeting and uptake remain to be fully elucidated.

## Exosomes and fibroblasts in PDAC stroma

**Fig. 1:**
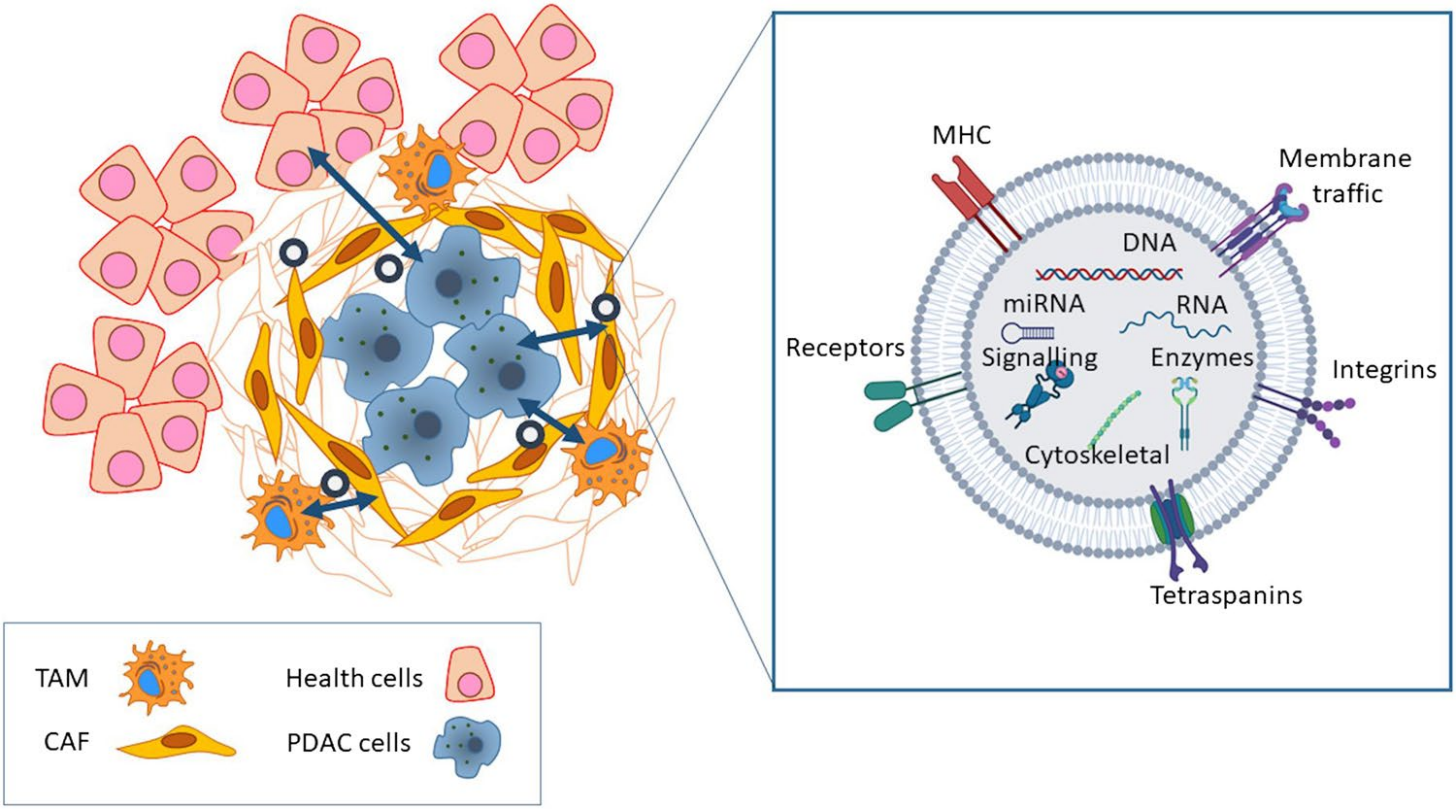
Exosomes are involved in the interactions between all the cell types present in the pancreatic tumor stroma. Interactions include the transfer of enzymes, signaling proteins, RNA, miRNA, and fragments of DNA. TAM, tumor-associated macrophages; CAF, cancer-associated fibroblasts; PDAC, pancreatic ductal adenocarcinoma

It is well known that in PDAC, the interactions between the stroma and the tumor cells define the behavior and characteristics of the tumor [16]. This means that there is a constant and very intense dialogue between the different cell types, and it is becoming increasingly clear that exosomes are very important actors in this dialogue (Fig. 1). In particular, the exchange of exosomes between tumor pancreatic cells and cancer-associated fibroblasts has been the subject of much research. In the specific case of the PDAC, cancer-associated fibroblasts that produce desmoplastic stroma originate from the activation of pancreatic stellate cells (PSCs)[35]. It has been reported that exosomes released by CAF play a relevant role in maintaining and promoting the tumor through different mechanisms.

The exosome-mediated signaling between pancreatic cancer cells and CAF is also involved in the resistance to gemcitabine, the standard chemotherapeutic agent for adjuvant therapy of PDAC. The mechanisms of action for gemcytabine are not completely understood. The main effect is the inhibition of DNA synthesis [42], but it also triggers apoptosis in response to cellular stress in tumor cells through the activation of p38 mitogen-activated protein kinase [12] and stimulates endogenous free radical’s generation by increasing NADPH oxidase activity [21]. Both PSCs and CAF are resistant to gemcitabine, and this chemoresistance could be transferred to pancreatic cancer cells through exosomes. This fact seems to be mediated by miR146a, which is induced in CAF by gemcitabine and increases its concentration in exosomes released by CAF exposed to this drug [41]. Another miR involved in this process is miR106b, that is also upregulated in CAFs and CAFs-exosomes after gemcitabine treatment and acts on pancreatic cancer cells by targeting TP53INP1, a key stress protein with tumor suppressor function [8]. Finally, it has also been reported the transfer of miR155 that acts downregulating the gemcitabine-metabolizing gene deoxycytidine kinase and, simultaneously, upregulating the ROS-detoxifying superoxide dismutase and catalase. In this case, upregulation results from the exosome-mediated transfer of their transcripts [37]. It should be noted that there are probably additional miRNAs transported carried by exosomes that are involved in the transfer of resistance to gemcitabine. For example, in lung cancer, it has been described that gemcitabine resistance is transferred by exosomes from resistant cells via delivery of miRNA-222-3p [46]. Since miRNA-222-3p levels are associated with a poor prognosis in pancreatic cancer [48], it is very likely that a similar mechanism of resistance takes place in PDAC stroma.

## Exosomes and tumor-associated macrophages in PDAC

Macrophages are also an important cellular component of PDAC stroma. Macrophages roughly could be classified into two main groups with different and sometimes opposite functions called classically activated macrophages (M1), involved in the inflammatory and antitumoral response, and alternatively activated macrophages (M2) that participate in immunosuppression and reparative processes. The stromal microenvironment, with its particular combination of cytokines, growth factors, anoxia, or lactic acid, causes macrophages to become tumor-associated macrophages (TAMs) with an M2 phenotype[58]. This phenotype confers resistance to tumors, and the presence of these TAM is associated with poor clinical outcomes [19]. They aid tumor progression by a number of mechanisms, including the expression of different growth factors, promotion of new vessel formation, immunosuppression, and enhancement of tumor motility through the production of matrix-remodeling enzymes [5].

As expected, exosomes play a prominent role in both the acquisition of the M2 phenotype and the pro-tumoral effects of TAMs. It has been reported in in vitro studies that the exosomes released by pancreatic cancer cells switch non-polarized THP1 macrophages to the M2 phenotype, thus inducing an increase in the secretion of pro-tumoral molecules including vascular endothelial growth factor, monocyte chemotactic protein-1, interleukin 6, interleukin 1β, matrix metallopeptidase 9, and tumor necrosis factor α [27]. On the other hand, KRAS^G12D^, the mutated form of KRAS associated with PDAC, has been shown to mediate the pro-tumorigenic M2 macrophage polarization. This KRAS^G12D^ can be transferred to macrophages by exosomes generated by PDAC tumor cells [7]. The fact that the abundance of KRAS^G12D^ in TAMs has been associated with poor prognosis of PDAC patients makes, again, this transport system a promising therapeutic target.

TAM also promotes tumor growth by a number of exosome-mediated mechanisms, usually involving different miRNAs. They induce angiogenesis by the inhibition of E2F2 expression in endothelial cells. E2F2 is a transcription factor involved in the impairment of the angiogenic response, and its inhibitions result in endothelial cell growth and angiogenesis[59]. In PDAC, the inhibition of E2F2 is achieved due to miR-155-5p and miR-221-5p carried by TAM-derived exosomes [50]. On the other hand, TAM could promote tumor cell growth through the inhibition of transforming growth factor beta receptor III (TGFBR3), a member of the TGF-beta superfamily coreceptor that functions as a tumor suppressor in pancreatic cancer [9]. It has been reported that TAM-derived exosomes transfer miR-501-3p to tumor cells, thus targeting TGFBR3 and facilitating the development of PDAC by activating the TGF-β signaling pathway [52]. Finally, as occurs with CAFs, TAM also could transfer chemoresistance to gemcitabine to pancreatic cancer cells through exosomes. This effect was mediated by the transfer of miR-365 that upregulates cytidine deaminase, the enzyme responsible for metabolizing gemcitabine to its inactive form [3].

Identifying the microRNAs involved in the different processes associated with PDAC offers a number of interesting therapeutic targets. Nevertheless, it should be considered that it may be more effective to find a way to control the flow and interactions of exosomes present in the PDAC stroma. Doing so would allow acting simultaneously on many pro-tumoral pathways. In this case, however, it should be taken into account that exosomes are complex systems that also carry microRNAs and other factors with antitumor activity. So, the balance between all these factors must be kept in mind when designing antitumor therapies based on these vesicles.

## Metastasis-promoting effects of stromal exosomes

As indicated, different proteins are considered constitutive markers of exosomes. This is related to the endosomal origin of these vesicles and includes different tetraspanins and annexins. Interestingly, some effects of these proteins result in structural changes in the stroma that potentiate the metastatic capacity of PDAC. It has been observed that Annexin A1 (ANXA1) present in exosomes can stimulate the activation of formyl peptide receptors, thus increasing cell motility on both fibroblasts and endothelial cells [34]. This mechanism requires the presence of ANXA1 on the external side of the exosomal membrane to allow its direct interaction with the formyl peptide receptors. This is an interaction similar to that described in some cancer models in which some tetraspanins, also present in exosome membranes, directly promote matrix degradation and reprogram stroma to a motile phenotype [54]. Several tetraspanins have been reported to act as metastasis-promoting mediators in different tumor systems. Interestingly, tetraspanins CD151 and Tspan8 present in exosomes could stimulate epithelial-mesenchymal transition. Exosomes interact with extracellular matrix proteins and contribute to matrix degradation in a mechanism mediated by the associated with proteases that results in an increased tumor and host cell motility [53].

## Interactions of stromal exosomes with healthy cells

Peri-tumor area, formed by healthy tissue surrounding the tumor mass, exhibits functional alterations related to the physical contact with the tumor cells as well as by the response to the bioactive mediators present in the stroma. Gene expression of these cells is closely similar to that induced in situations of cell stress. A good example is the expression of Reg3β, a 16 kDa secretory protein also known as pancreatitis-associated protein (PAP), intensely expressed during acute pancreatitis that, in the case of PDAC, has been shown to be expressed but only in those areas of healthy pancreatic tissue that are in contact with the tumor [28]. The release of Reg3β into the tumor stroma impacts the behavior of the exosomes present in that area, as Reg3β adheres to the surface of the exosomes, thus interfering with their possible interactions with potential target cells [4]. This fact suggests the existence of a gradient within the tumor stroma in which the role of exosomes may experience functional differences, depending on how far apart they are from the boundary zone between tumor tissue and healthy tissue[20].

## Exosome-based therapies

As soon as the role of exosomes in the pathogenesis of cancer began to be understood, it became clear that these vesicles offered enormous potential as biomarkers and in the design of different therapeutic approaches. Some studies have shown that different microRNAs carried by exosomes can be used as PDAC early markers. For instance, miR-196a and miR-1246 or miR-451a were found to be highly expressed in exosomes derived from pancreatic cancer [10, 49]. On the other hand, exosomes have been assayed as a delivery system for chemotherapeutical drugs. Different preclinical assays have been reported administering exosomes loaded with oxaliplatin or gemcitabine but also with different microRNA or biomolecules that increase cytotoxicity as curcumin [2, 36, 61]. Finally, exosomes were also engineered to carry siRNA or shRNA specific against the oncogenic KRAS^G12D^, the most common mutation in pancreatic cancer [24].

## Conclusions

The pancreatic tumor stroma is an area with very extensive exchange of signals between different cell types. This network of interactions will greatly condition the behavior of the tumor, its aggressiveness, its growth, and the ease with which it will generate metastases in distant places. For some years now, exosomes have been shown to be important players in the exchange of signals that take place in the tumor stroma. Many of the characteristics that make pancreatic cancer such an aggressive pathology have some of its basis in the role of exosomes. For this reason, these vesicles are being placed in the spotlight in order to build therapeutic strategies aimed at blocking or redirecting the signals sent by these signal exchangers.
